# Medicine and the War

**Published:** 1904-02-20

**Authors:** 


### The Hospital.

A Journal of

The Medical Sciences and Hospital Administration.

Vol. XXXV.—No. 908. FEBRUARY 20, 1904.

### Medicine and the War.

Few things will be more interesting, in the great struggle which is commencing between Japan and Russia, than the provision which will be made by the respective belligerents for the preservation of the health of the soldiers, and for the care of the sick and wounded. It seems improbable that any accurate information upon the subject will be available from either side while the war is actually in progress, because so far no permission has been given for representatives of the European press to accompany the armies, and because the commanders on both sides are likely to be too well aware of the advantages of secrecy, and too indifferent to the craving of foreigners for news, to render it in the least degree probable that the existing prohibitions will be relaxed. At the close of the struggle, however, we may reasonably expect to be furnished by Japan with a scientifically correct account of all the conditions of the strife, as they were seen from her own points of view; and we shall probably find that she has fully utilised the experience of past conflicts to secure all possible advantages to those fighting under her flag. In Japan, as in China, the value set upon human life, as such, is said to be comparatively small; but against this there will be the unquestionable importance of every unit in a struggle against a Power apparently capable of bringing larger numbers into the fighting line, and of replacing inevitable losses from a larger and individually less valuable population. In these circumstances a single soldier is worth more to Japan than to Russia, and the care taken for his protection is likely to be determined accordingly. We know that Japan has physicians and surgeons second to none; and we have every reason to believe that her military system is free from the abuses, in the shape of routine and of the influences of what is called society, which are so powerful in retarding improvement, and in promoting incompetency, in many other countries. We shall fully expect to hear that everything possible was done to guard against that most terrible scourge of armies, enteric fever. If the medical advisers of the Commander-in-Chief declare boiled water to be a necessity, a reasonable attempt will be made, no doubt, to supply the want; and the result, whatever it may be as far as the forces in action are concerned, will be hereafter available for the guidance of all other countries. In like manner the organisation of the field hospitals will probably be controlled by the most recent experience of other campaigns, and notably by our own in South Africa, with the probable result that many of the mistakes incidental to inexperience will be either minimised or avoided. We may presume that Japan contains no "little Japanese," that there will be no representative of Sir Henry Campbell-Bannerman to attribute methods of barbarism to her soldiers, and no representative of Mr. Burdett Coutts to describe her field hospitals; but we may reasonably expect that the absence of these advantages will find compensation in the intense patriotism certain to be developed by what is practically a national struggle for existence, and which will seek to secure the highest efficiency in every branch of her naval and military service. Her representatives are making history, and they are practically certain to spare no pains in the effort to accomplish the arduous task which they have undertaken.

With regard to Russia, we have as little information as with regard to Japan. It was recently reported that her men were "dying like flies" in Manchuria, and this is about equally likely to be the truth, or to be a falsehood put into circulation for ulterior ends. All we know is that the forces at Port Arthur and along the Yalu River are a great distance from their base of operations, and are connected with it by a single line of railway which was constructed with great rapidity, and therefore, in all probability, not in the best and most secure manner. Our own experience in South Africa throws considerable light upon the difficulty of supplying and reinforcing an army thus situate. It would be delusive to argue from the conduct of Russia during the Crimean war to her probable conduct in the present one; because we ourselves have learnt much in the course of the intervening time, and she may possibly have done the same. We have absolutely no information from either side as to what may very possibly be, after all, the chief determining factor in the strife, that is to say, the individual capacities of the respective leaders, most of whom have still to show of what they are capable. Sebastopol would have fallen a week after the battle of the Alma if it had not been for the previously unsuspected genius of Todleben; and the issues of war in the Gulf of Pe-che-li may depend upon whether or not Port Arthur finds a capable defender. It is by no means impossible that the recent persecutions of the Jews in Russia may have the effect of enlisting capitalists on the side of her adversary; and, if so, the "sinews of war" may, after all, be the chief determining factor in the result. Whatever this result may be, it will assuredly be for the benefit of mankind that the progress of Japan should not be too rudely or too seriously arrested, and that she should be left free, at the termination of the strife, to pursue unchecked that course of national development and of progressive civilisation which have already made her one of the wonders of the world.

